# Pneumatosis: Appearances on CT Imaging

**DOI:** 10.7759/cureus.41927

**Published:** 2023-07-15

**Authors:** Daphne J Theodorou, Stavroula J Theodorou, Yousuke Kakitsubata

**Affiliations:** 1 Radiology, General Hospital of Ioannina, Ioannina, GRC; 2 Radiology, Miyazaki Konan Hospital, Miyazaki, JPN

**Keywords:** accidental pneumoperitoneum, epidural pneumorrhachis, spontaneous pneumomediastinum (spm), emphysema, peritoneum, spinal, mediastinum, pneumatosis

## Abstract

Background:Pneumatosis is a general term used to designate the presence of spontaneous air or gas leaks into the body's compartments.

Purpose: In this paper, we provide an overview of gas originating from different sites and present the most common routes by which air may escape free to surrounding or distant tissues.

Methods: On the basis of 45 interesting clinical cases, we discuss the CT imaging characteristics of thoracic and spinal pneumatosis, better known as pneumomediastinum and pneumorrhachis. In addition, we present craniocervical pneumatosis manifesting as subcutaneous emphysema.

Results: Isolated pneumatosis was diagnosed in 12 (27%) of the 45 patients, manifesting as craniocervical free air or pneumoperitoneum. In 28 (62%) patients with pneumomediastinum, 12 (43%) had concomitant pneumothorax. Soft tissue emphysema was seen in 24 (52%) patients. One of the patients with generalized pneumatosis had craniocervical and extensive soft tissue emphysema, in conjunction with pneumomediastinum, pneumothorax, and pneumoperitoneum. Intraspinal pneumatosis was always coupled with pneumomediastinum.

Conclusion: Pneumatosis may not be as uncommon as it seems, and indeed, this condition may need to be recognized early as it can be an alarming sign of serious pathology.

## Introduction

Although rare air or gas leaks into the body compartments can occur spontaneously, various well-recognized conditions can be associated with free air (pneumatosis) released from the lungs, the airways, the abdomen, or the spine. Medical procedures, blunt trauma, unaccustomed positive pressure activities, and infection are among the conditions associated with pneumatosis in the craniocervical and thoracic areas and the spine.

Indeed, the collection of free air or other gas in the cervicothoracic region and the spinal epidural space has been sporadically reported in the literature in association with numerous conditions. Included among them are fractures, surgical procedures, bronchial asthma, strenuous cough, underwater diving, inflammatory bowel disease, and diabetic ketoacidosis [1-5]. By the time free air is released, it dissects through tissue planes to form collections, or it can travel to other adjacent or distant anatomic sites, causing symptoms. Because pneumatosis may appear isolated or in conjunction with other disorders, it is important to evaluate the presence of free air, which may have implications for the etiology and pathological mechanisms underlying this serious disease. On radiographs, air is radiolucent, and as such, it can be readily apparent on good-quality views. Similarly, gas has a Hounsfield unit number less than zero, and it can be readily seen on computed tomography (CT) images. Further, CT scans can easily detect small foci of gas or more extensive pockets of air that may have accumulated in a single body compartment or at different sites. Magnetic resonance (MR) imaging may not be required to detect free air in most cases of pneumatosis; however, major indications for MR imaging in cases of suspected pneumatosis involve the detection of air in the spine. Radiologists come across cases of pneumatosis in routine practice.

Because of complicated cases that are usually related to high-energy trauma, radiologists need to be vigilant in exploring more than meets the eye, as free air can reside in distant sites other than the readily obvious ones. The purpose of this study is to address the wide spectrum of imaging findings in pneumatosis across the body and illustrate cases we and all practicing radiologists may come across on an emergency basis.

## Materials and methods

Patients with pneumatosis who had undergone CT or MR imaging examinations were identified by the authors at two institutions: the Department of Radiology at the General Hospital of Ioannina, Ioannina, Greece, and the Department of Radiology at Miyazaki Konan Hospital, Miyazaki, Japan. The patients' imaging findings were evaluated by three board-certified radiologists acting in consensus.

The study group comprised 45 patients with pneumatosis (37 men and eight women; the age range of the participants was between 18 and 82 years). Patients were included in the study group if they fulfilled the following criteria: i) The diagnosis of pneumatosis could undoubtedly be made on conventional radiography or cross-sectional imaging studies; ii) The imaging studies were available for review. With regard to the first criterion, the imaging examinations were reviewed for the presence of free air and other findings that may be important in the evaluation of pneumatosis, including the location of gas, the presence of a fracture or other trauma, a tumor, deep or soft-tissue infection or inflammation, and no obvious pathology in those cases with spontaneous pneumatosis. Patients were excluded from the study if the clinical or surgical data suggested a diagnosis other than pneumatosis.

The age, gender, history, and clinical data were recorded and categorized. The patients presented with histories of blunt trauma (n = 30), possible fracture (n = 7), infection (n = 3), inflammation (n = 1), possible tumor (n = 1), and no obvious pneumatosis-related pathology (n = 4). The data were then analyzed, and because of the small number of cases, there was no formal statistical analysis.

## Results

All patients were symptomatic upon presentation to the emergency departments of the two hospitals. Local pain and swelling, soft tissue crepitus, and dyspnea were common findings.

Six patients underwent surgery with a diagnosis of gas gangrene (n = 3), fracture(s) (n = 2), and tumor (n = 1). Radiography was diagnostic of pneumatosis in 42 patients; three patients with intraspinal air were not diagnosed by using radiography. The CT images were positive for pneumatosis in all 45 cases. MR images were obtained for four patients and detected free gas in the spinal canal (n = 3) and the neck region (n = 1). Of 45 patients, isolated pneumatosis was recorded in 12 (27%) instances as follows: craniocervical (n = 8) and pneumoperitoneum (n = 4). Pneumomediastinum was seen in 28 (62%) patients, and it was associated with pneumothorax in 12 (43%) of these cases. Soft tissue emphysema was quite common and was seen complicating other forms of pneumatosis in 24 (52%) patients. One of the patients with generalized pneumatosis had craniocervical and extensive soft tissue emphysema, in conjunction with pneumomediastinum, pneumothorax, and pneumoperitoneum. In all three patients with intraspinal pneumatosis, a concomitant pneumomediastinum was present.

## Discussion

Pneumatosis: air all over

**Figure 1:**
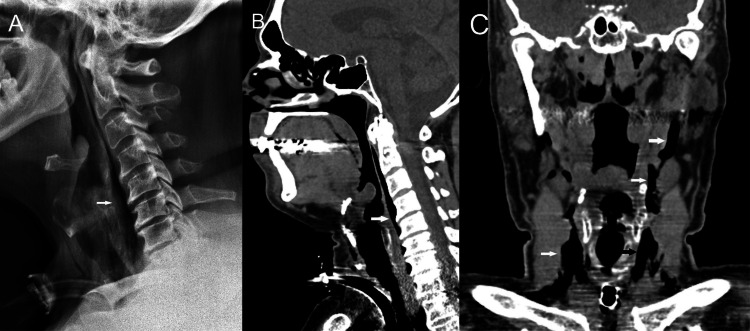
Scans of a 61-year-old man intubated due to a serious COVID-19 lung infection The lateral radiograph (A) and reformatted sagittal (B) and axial (C) CT images show craniocervical pneumatosis (arrows).

**Figure 2:**
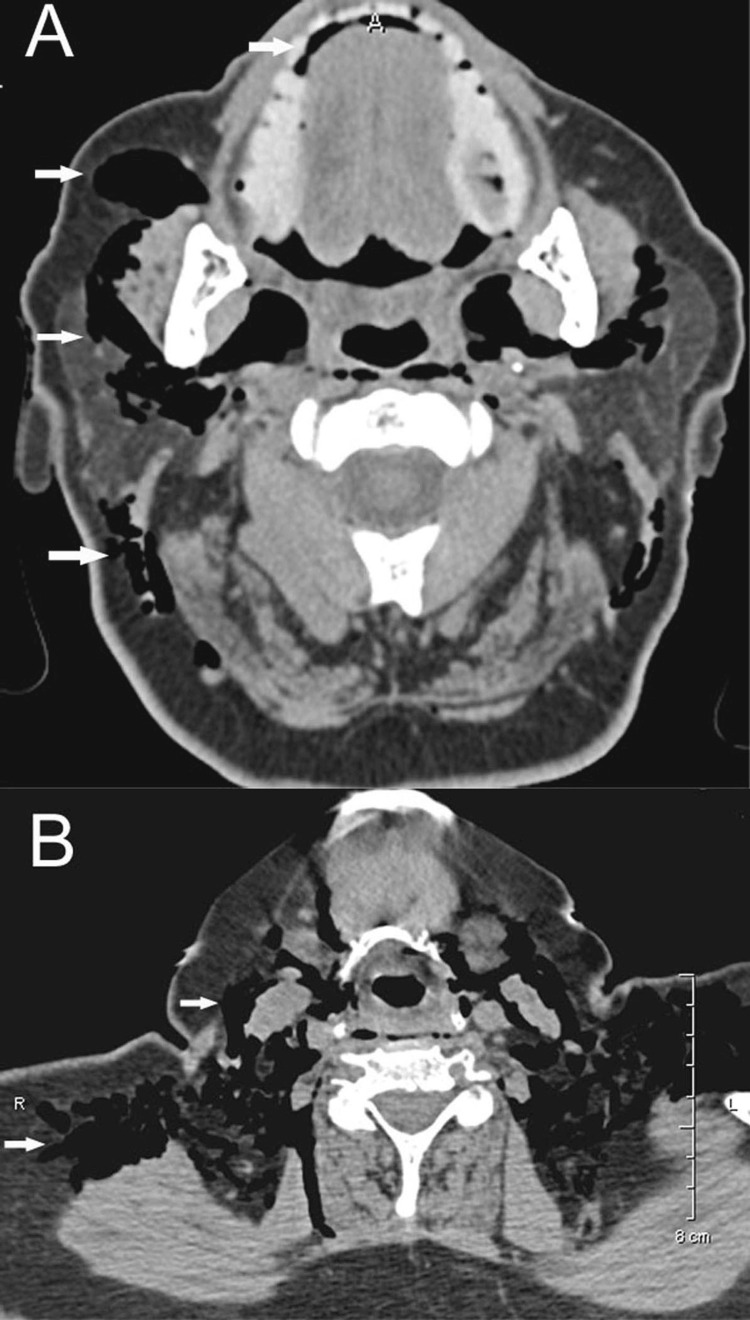
Scans of an 81-year-old woman who committed suicide with a knife, stabbing her face and neck Axial CT images (A) and (B) depict multiple pockets (arrows) of craniocervical and cervicothoracic free air.

**Figure 3:**
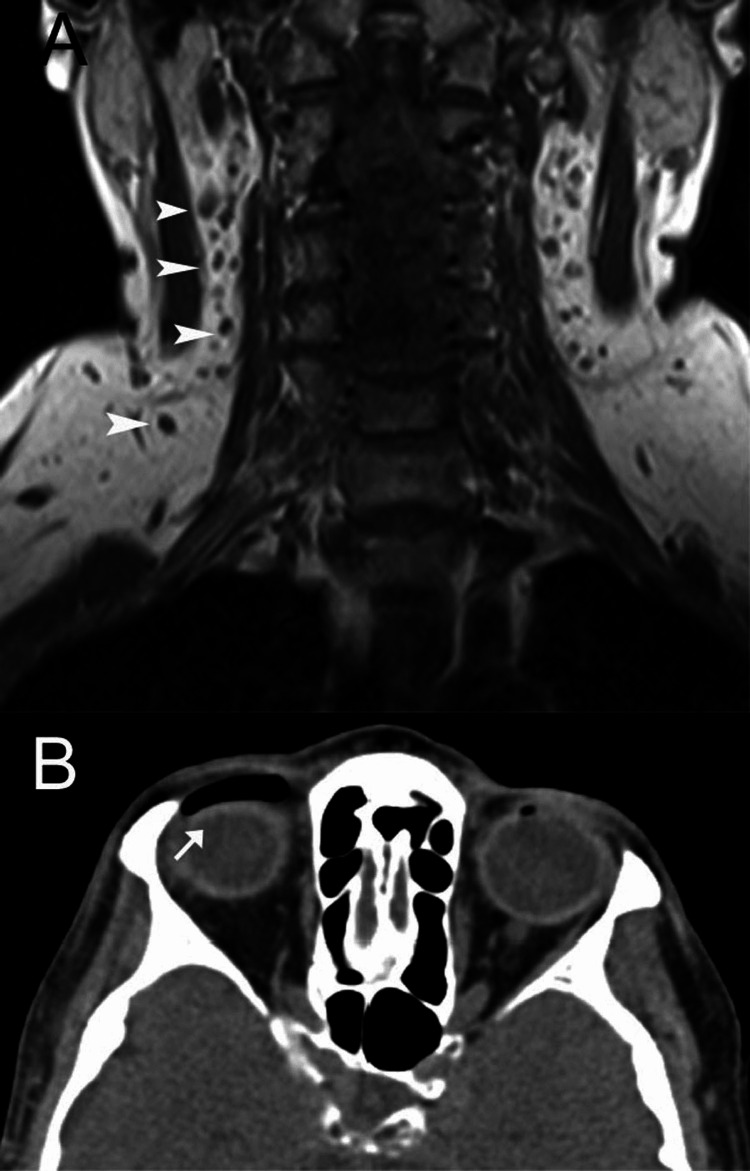
Scans of a 72-year-old woman who developed craniocervical pneumatosis after a tooth extraction A: The coronal T1-weighted MR image shows numerous air bubbles (arrowheads) in the neck; B: On the CT image, a pocket of air is seen in the upper lid (arrow).

Craniocervical or cervicofacial emphysema denotes the presence of air in the soft tissue of the head and neck. Trauma, surgery, anesthesia, coughing, and habitual performance of the Valsalva maneuver have a reported causal relationship with the introduction of free air in soft tissue. There are numerous reports of subcutaneous emphysema developing after dental extractions performed with high-speed air turbine drills [2, 6]. Emphysema usually propagates to the neck and the mediastinum, comprising cervicothoracic pneumatosis and pneumomediastinum, respectively [1, 2] (Figures 1-3).

**Figure 4:**
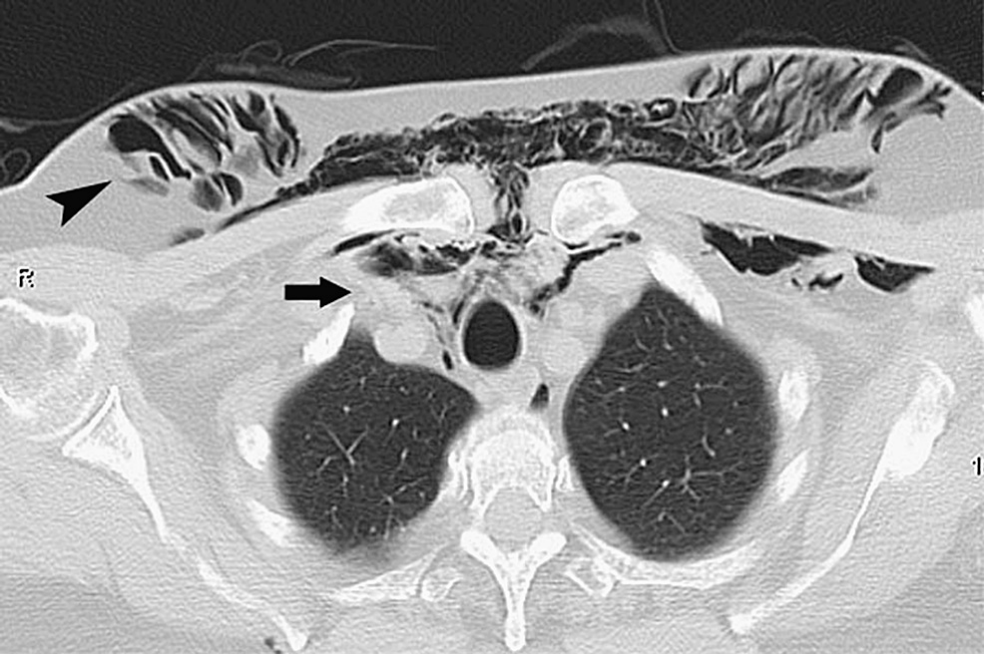
Scan of a 65-year-old woman who was stabbed with a knife by a thief There is extensive subcutaneous emphysema in the anterior thoracic wall and the breasts (arrowhead), as well as pneumomediastinum (arrow).

**Figure 5:**
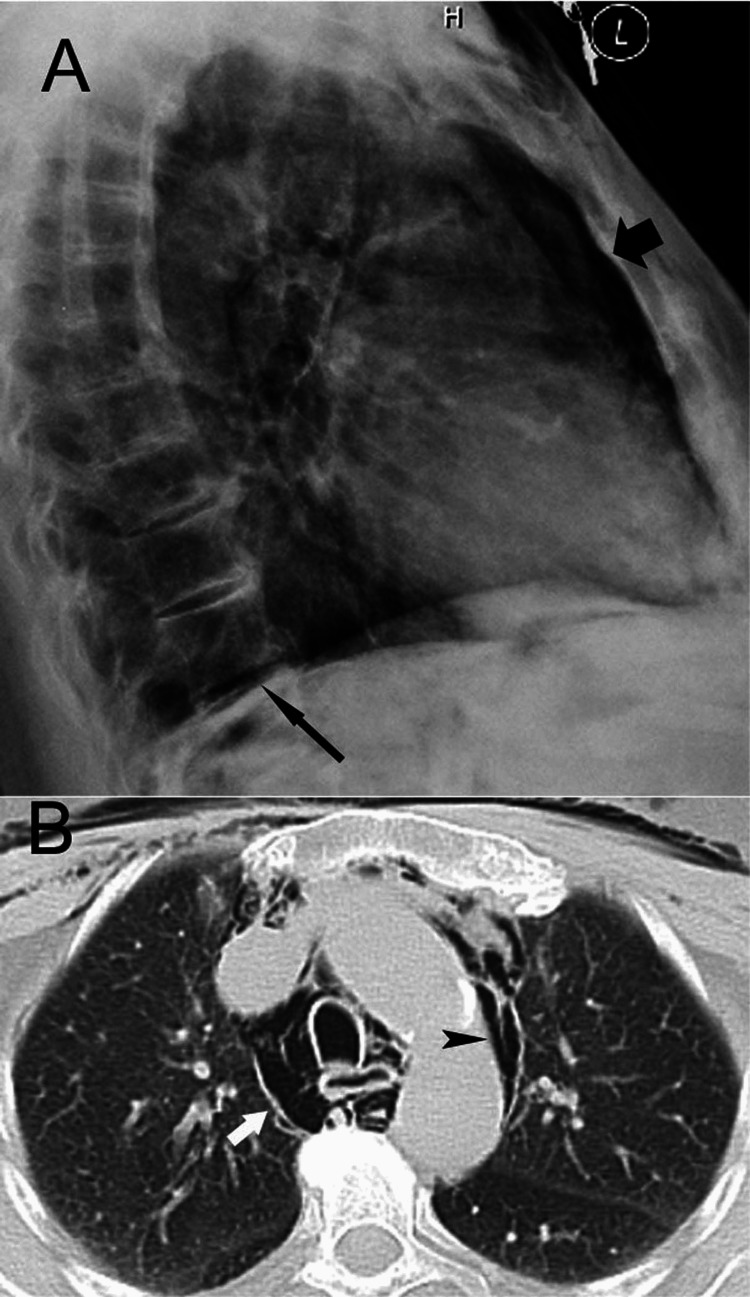
Scans of a 72-year-old female who underwent a colonoscopy that caused perforation of a diverticulum in the sigmoid A: A lateral chest radiograph shows pneumomediastinum (thick arrow) and pneumoperitoneum (long arrow); B: A CT image delineates pneumomediastinum (arrowhead) and pneumothorax (arrow).

**Figure 6:**
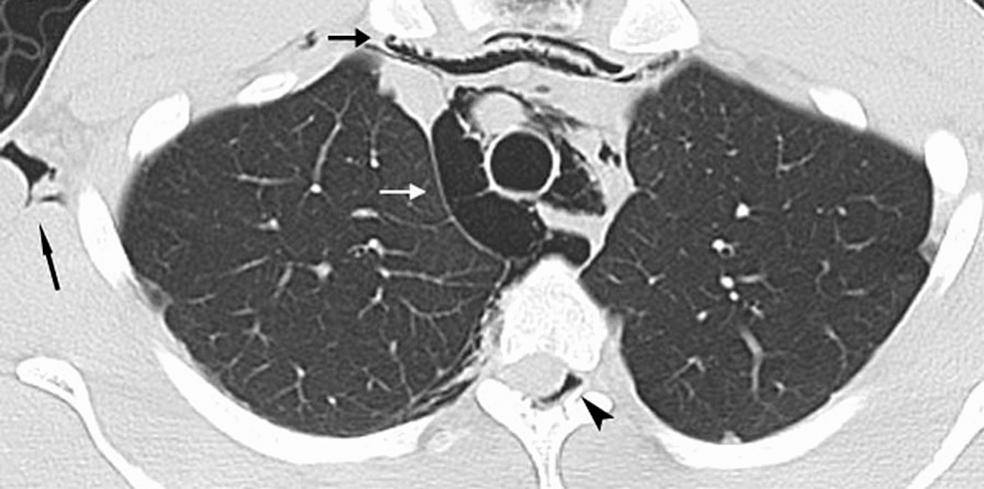
Scans of a 21-year-old male who developed spontaneous pneumatosis Air was released in the mediastinum (arrows), the thoracic wall (long arrow), and into the thoracic spinal canal (arrowhead).

**Figure 7:**
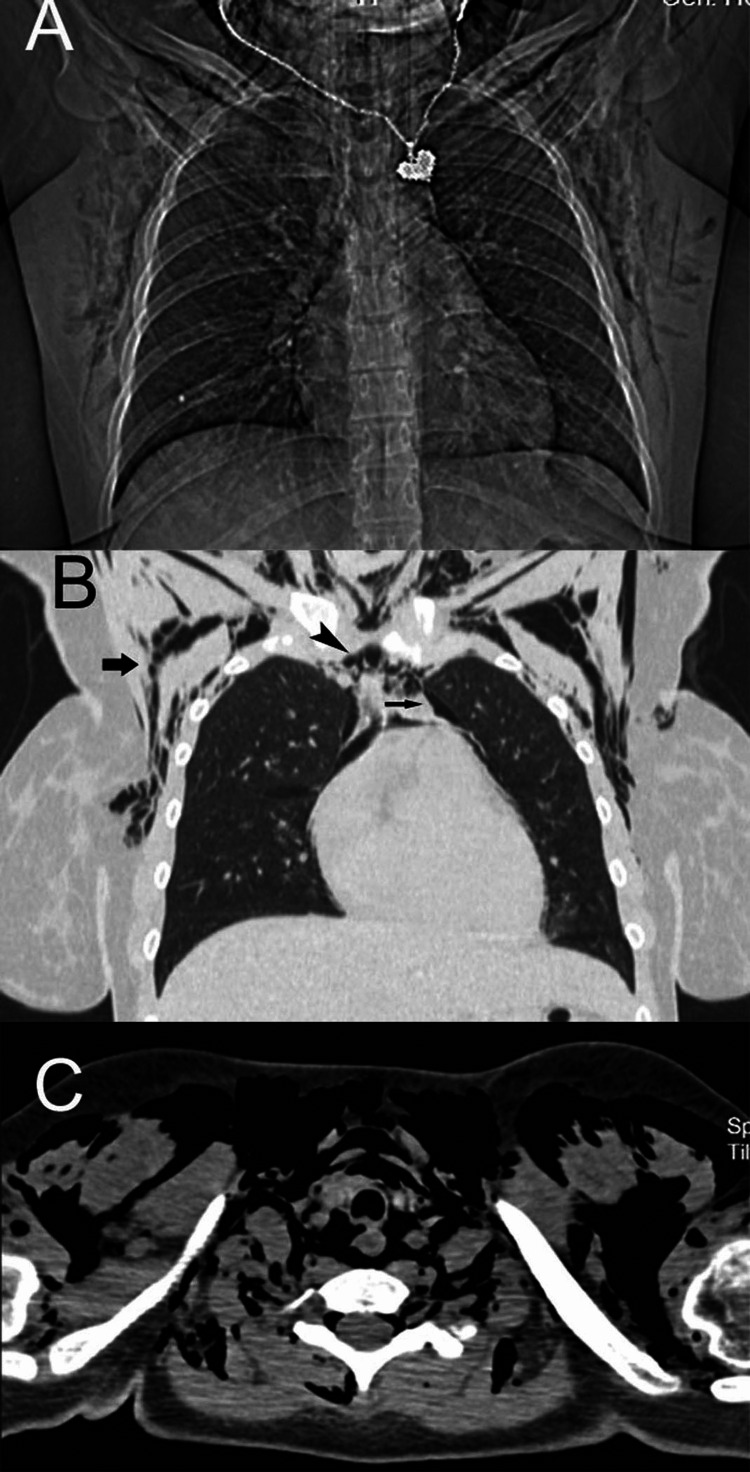
Scans of an 18-year-old female who developed pneumomediastinum, pneumothorax (arrow), pneumorrhachis, and subcutaneous emphysema with paroxysmal cough A: An anterior-posterior chest radiograph shows extensive soft tissue emphysema and a possible pneumothorax; B: A coronal reformatted CT image reveals pneumothorax (thin arrow), pneumomediastinum (arrowhead), and soft tissue emphysema (arrow) in the axillary regions and the neck; C: The axial CT image displays extensive emphysema. Free air resides in soft tissue, the mediastinum, and the spinal canal.

As per its name, pneumomediastinum, also known as mediastinal emphysema, is the collection of extraluminal air in the mediastinum. Abnormal gas escapes, causing increased intrapulmonary pressure, manifesting in chest pain and labored breathing. Other than spontaneously, pneumomediastinum may develop secondary to dental procedures, general anesthesia, illegal drugs, bronchial asthma, mediastinal infection, tumors, and diabetic ketoacidosis [7, 8]. Urgent surgical decompression is warranted if a large airway or cardiovascular collapse occurs. On chest radiography, an abnormal collection of air in the mediastinum can be seen outlining the heart. Computed tomography images provide a more detailed analysis of the exact sites of the air pockets forming around the major airways, the heart, and the vessels. Pneumomediastinum may coexist with pneumothorax and usually resorbs slowly (Figures 4-7).

**Figure 8:**
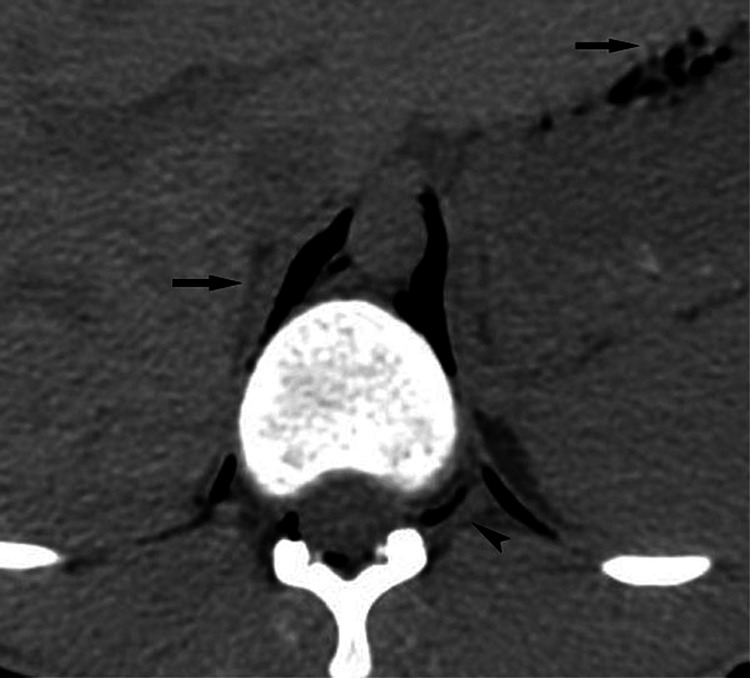
A scan of a 23-year-old man who developed spontaneous cervicothoracic emphysema and pneumomediastinum Free air (arrows) is seen tracking along the diaphragmatic crura and communicating with the peritoneum. There is air dissecting in the spinal canal (arrowhead), comprising pneumorrhachis, and coursing along the nerve root sheath.

By definition, pneumorrhachis is the collection of air in the spinal canal. Free air may be epidural or subarachnoid. Pneumorrhachis can be benign and self-resolving, or it can have an unfavorable prognosis (Figure 8).

**Figure 9:**
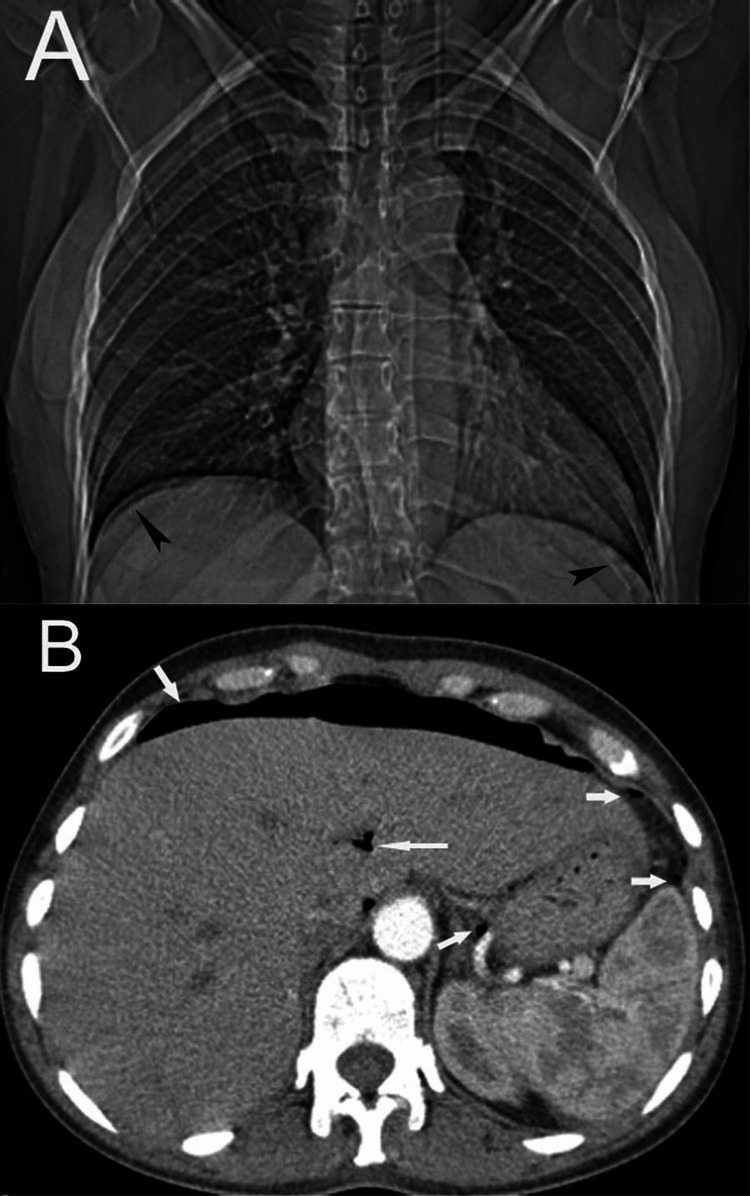
Scans of a 48-year-old female who developed pneumoperitoneum (arrows) during hysterectomy A: The frontal chest radiograph reveals subdiaphragmatic free air (arrowheads); B: An axial CT image of the abdomen displays free peritoneal air (arrows). Free air is also visualized in the biliary tract (long arrow).

**Figure 10:**
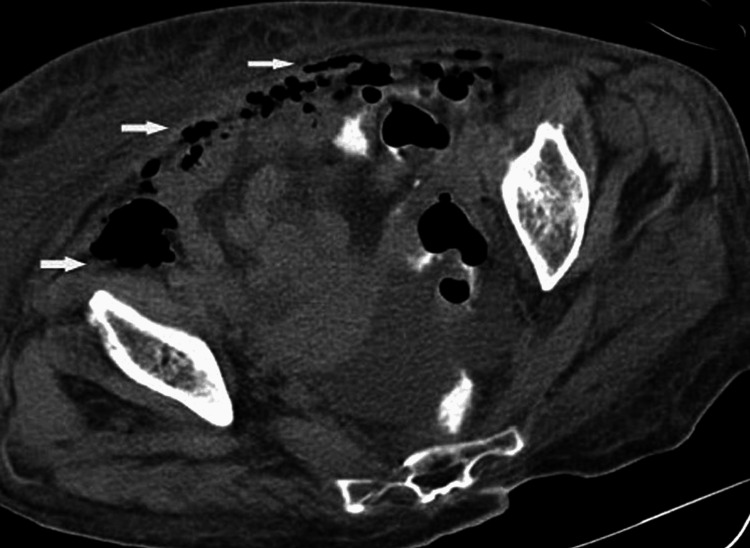
Scan of a 70-year-old female who developed diabetic ketoacidosis An axial CT image shows free air (arrows) in the abdomen and pelvis. Extensive anasarca edema is present.

Either spontaneous or associated with pneumomediastinum in many instances, pneumorrhachis has been related to a broad spectrum of abnormal conditions, including spinal trauma or infection [3-5, 9]. Pneumoperitoneum is defined as the abnormal presence of air in the peritoneal cavity that may result from tissue ischemia, visceral perforation, infection, or mechanical or thermal injury. Cancer, iatrogenic injury, infection, and ulcerative disease are predisposing factors for serious morbidity [10-13] (Figures 9, 10).

**Figure 11:**
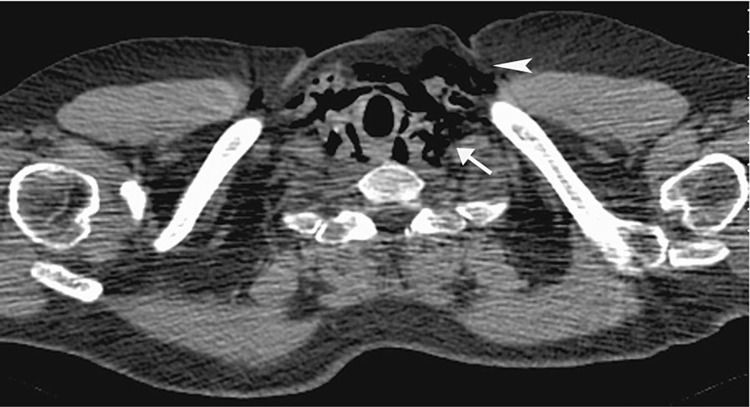
Scan of a 63-year-old woman with spontaneous pneumomediastinum (arrow) and subcutaneous emphysema in the neck (arrowhead)

The release and entrapment of free air in subcutaneous soft tissues are known as subcutaneous emphysema. The condition is not uncommon and is usually self-limited [14, 15] (Figure 11).

**Figure 12:**
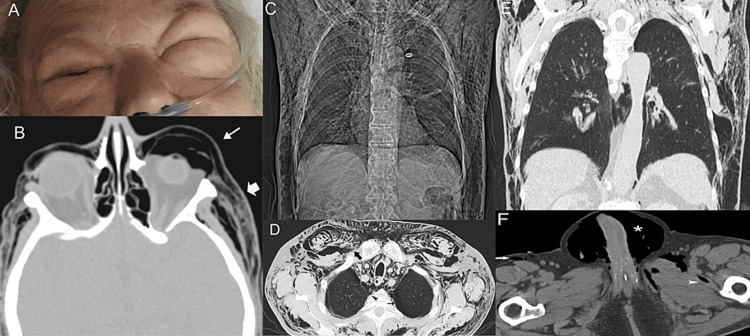
A 62-year-old man developed generalized pneumatosis after falling off a boat flagpole. Free air is seen literally from head to toe. A: A photograph of the patient shows facial pneumatosis; B: An axial CT image through the orbits shows emphysema of the upper lid (arrow) and the temporal region (thick arrow), on both sides; C: A coronal scout CT image shows marked pneumatosis occupying soft tissues of the lower neck, thorax, and upper abdomen; D: An axial CT image of the chest reveals pneumomediastinum (arrowhead), pneumothorax (arrow), and soft tissue emphysema (thick arrow); E: A coronal reformatted CT image of the chest shows pneumatosis; F: An axial CT image through the pelvis reveals marked scrotal emphysema (asterisk). Note free air in the subcutaneous soft tissue (arrow) and the intermuscular fascia (arrowhead).

Finally, massive generalized emphysema may occur in patients who have sustained serious trauma or infection and in the population of older people (Figure 12).

Radiologists need to maintain a high index of suspicion in cases where the possibility of the release of free air fails to come to clinical attention. Air leaks may be localized at the site of primary pathology or may dissect through different tissue planes and unexpectedly appear at distant sites as well. For example, after a forceful cough, free air from the ruptured alveolar space may produce pneumothorax, pneumomediastinum, or pneumopericardium, or the released air while traveling along the fascial planes may reach the neck (i.e., submandibular and retropharyngeal space). Following the periaortic and peri-esophageal fascial planes, air may present within the peritoneum or retroperitoneum, causing pneumoretroperitoneum and soft tissue. Surprisingly enough, free air may dissect the path of neural foramina and accumulate in the epidural space. Apparently, the combinations of pneumatosis within different tissues and organ systems are endless. In our practices, and especially on an emergency basis, we have come across many cases of pneumatosis. Either isolated in one major system or involving different ones, pneumatosis implies serious nosology. Radiography may readily depict free air, although the exact location and distribution of the free gas collections will need to be explored in CT studies. MR imaging is reserved for those cases with free air queried for intraspinal pathology. This collective work aims to present clinical cases that radiologists may come across in routine clinical practices. As said, we have encountered these cases on an emergency basis and think that they merit reporting because the CT imaging presentations are intriguing, and efficient reporting of the imaging findings can have a substantial impact on prompt diagnosis and patient management.

## Conclusions

In diagnosing pneumatosis, radiography is the initial and readily available imaging method for the direct depiction of free air in the body. Computed tomography is of additional diagnostic value as it allows for the detection of free air in a specific body compartment or soft tissue, providing the exact site and extent of the collection of gas. Most importantly, CT allows the assessment of the involvement of adjacent organs and can document associated abnormalities, such as trauma or serious infection. Assessment of the spinal canal and the spinal cord is enabled by MR imaging.
